# A blood-based transcriptomic signature stratifies severe Crohn’s disease and defines potentially targetable therapeutic pathways

**DOI:** 10.3389/fgstr.2023.1251133

**Published:** 2023-10-18

**Authors:** Rivkah Gonsky, Evan Adams, Alka A. Potdar, Gregory Botwin, Eva Biener-Ramanujan, Dermot P. B. McGovern, Jonathan G. Braun, Phillip Fleshner, Stephan R. Targan

**Affiliations:** ^1^ F. Widjaja Inflammatory Bowel Institute, Cedars-Sinai, Los Angeles, CA, United States; ^2^ Division of Colorectal Surgery, Department of Surgery, Cedars-Sinai Medical System, Los Angeles, CA, United States

**Keywords:** Crohn’s disease, transcriptomic signature, disease subgroup stratification, biomarker, gene regulation

## Abstract

**Introduction:**

Despite advances in medical therapy, many patients with Crohn’s disease (CD) ultimately require surgery for disease management. Identifying the underlying molecular pathways for subgroup stratification is critical to the improvement of prognostics and therapeutics and to biomarker discovery.

**Methods:**

We purified CD3^+^ T cells from the paired blood and mucosa samples of 100 CD and 17 non-inflammatory bowel disease (IBD) subjects requiring surgery. Longitudinal samples (*n* = 49) were collected 4–13 months postoperatively.

**Results:**

Transcriptional profiling at the time of surgery revealed two CD patient subgroups: the CD-PBT subgroup, which was clustered tightly with non-IBD subjects, and the CD-PBmu(cosal) subgroup, which shifted from peripheral toward a mucosal-like expression profile. The CD-PBmu subgroup was characterized by differential gene expression, elevated genetic transcriptional risk score (TRS), and a distinct T-cell subset composition associated with perianal-penetrating/stricturing disease, post-surgical recurrence, and immunoreactivity to multiple microbial antigens. CD-PBmu subtyping was validated in a CD cohort in whom anti-TNF therapy had been unsuccessful. The CD-PBmu subgroup, in contrast to the CD-PBT subgroup, was distinguished by decreased pro-inflammatory cytokine/chemokine and adhesion molecule expression postoperatively. For clinical translation, we identified a CD-PBmu 42-gene classifier associated with a TRS signature, clinical severity markers, and underlying protein kinase signaling pathways to identify therapeutic targets.

**Discussion:**

The CD-PBmu signature holds potential for future investigation to improve accuracy in identifying a subset of patients with severe CD who may benefit from early initiation of therapeutics to defined molecular pathways.

## Introduction

Crohn’s disease is a clinically and biologically heterogeneous disorder characterized by chronic transmural inflammation of the gastrointestinal tract. Current treatment options frequently fail to initiate and sustain long-term remission. CD can present as mild, i.e., non-stricturing/non-penetrating, but it often progresses to severe, complicated disease (1, 2). Patients whose disease is refractory to therapeutic modulation or who exhibit complications often require surgery for disease management. The development of an index to clinically define the severity of CD has been useful (3, 4); however, currently there is no predictive model for the identification of patients in whom therapeutics are likely to be unsuccessful and who will ultimately require surgery(s). The lack of concordance between clinical remission and subclinical inflammation poses a challenge for medical management. Thus, novel approaches are needed not only to develop better prognostic biomarkers but also to identify distinct subpopulations that are likely to benefit from the development of new, more effective, personalized treatments that eradicate or mitigate disease complications.

Recent research efforts have focused on developing CD biomarkers to predict disease course, drug response, and clinical outcomes. For example, expression signatures and genetic associations have added to our understanding; however, they explain only a small proportion of overall disease heterogeneity. The vast majority of these studies have focused on identifying factors driving disease progression when comparing patients with active CD with control subjects, or with patients with mild disease or those naive to treatment. Gene expression studies focusing on the population in which therapeutic intervention was unsuccessful and with resistant, complicated disease requiring surgical intervention are rare (5). Furthermore, few studies have identified biomarkers that are predictive of clinical outcome or that further illuminate the nature of inflammatory bowel disease (IBD) biology in the gut. Understanding the underlying pathobiology involved in this medically challenging CD patient group could lead to the discovery of patient subtype-targeted therapeutics and to enhanced treatment efficacy.

In this study, we examined gene expression profiles within matched mucosal and circulating T cells from CD patients with refractory disease that were obtained at the time of surgery for disease management. We show severe CD can be stratified into two distinct subtypes based on peripheral blood (PBT) T-cell gene expression: (1) the subtype CD-PBT, in which circulating peripheral T cells are present; and (2) the subtype CD-PBmu (cosal), in which patient samples exhibit a mucosal cell-like transcriptomic profile, an elevated genetic transcriptional risk score (TRS) signature, and altered T-cell subset composition associated with clinical features of complicated disease compared with those of patients who had the CD-PBT subtype. A defining hallmark of the CD-PBmu subtype is the marked downregulation of pro-inflammatory cytokines, chemokines, and adhesion molecules following surgery. These findings raise the prospect of defining molecular subtypes within CD and have the potential to guide effective therapeutic regimens and improve clinical outcomes.

## Materials and methods

### Study subjects

Human subjects were recruited through the MIRIAD IBD Biobank at the F. Widjaja Research Institute at Cedars-Sinai, Los Angeles, CA, USA. Informed consent (approved by the Institutional Review Board at Cedars-Sinai) was obtained from all participating subjects. Clinical information was obtained from CD patients prior to their undergoing surgical resection and prospective follow-up. Non-IBD subjects had no known history of IBD and underwent surgery for cancer (5 out of 17 subjects), diverticulitis (4 out of 17 subjects), familial adenomatous polyposis (2 out of 17 subjects), polyps (3 out of 17 subjects), and other conditions (colonic inertia, trauma, or retained capsule, 3 out of 17 subjects). CD and non-IBD samples were collected from surgical resections that were carried out by a single provider. Disease recurrence was based on Rutgeerts endoscopic scoring, with a score of ≥2 indicating recurrence. A pre-operative Crohn’s disease severity score was calculated based on a modified disease severity weighted index (3). The attributes included fistula, perianal abscess, steroid use, biologics/immunologics use, stricture, and disease extent. Patients who had previous resections were assigned a weighted score of 3, and a score of 0 indicated no prior resection. All laboratory procedures, staff assessments of patient phenotypes, and gross pathological severity score calculations were conducted by staff blinded to the results of all *in vitro* assays. The criteria for gross pathological severity score diseased segments included the extent of the stricture, ulcer, fistula, and/or diseased mucosa. Subjects were stratified into three categories based on their pathologic features and the extent of their disease: mild (< 3cm), moderate (3–5cm), and severe (> 5cm, multiple fistula tracks, deep ulceration, and/or severe microscopic disease).

### Isolation of purified CD3^+^ peripheral and mucosal T cells

Blood and intestinal specimens were obtained from CD patients undergoing intestinal resection. PBMC were isolated by separation on Ficoll–Hypaque gradients. Lamina propria mononuclear cells (LPMCs) were isolated from the resection samples using a technique described previously (6). CD3^+^ T cells were isolated using CD3-immunomagnetic beads (Miltenyi Biotech, Auburn, CA), which enabled at least 95% of the pure CD3^+^ T cells to be isolated without T-cell activation.

### Gene expression assay for CD3^+^ T cells and whole blood

Whole RNA was extracted from CD3^+^ T cells and libraries for RNA-Seq were prepared using the Nugen human FFPE RNA-Seq library system. The workflow consists of cDNA generation, fragmentation, end repair, adaptor ligation, and PCR amplification. Different adaptors were used for multiplexing samples in one lane. Sequencing was conducted on an Illumina NextSeq 500 for a single-end read run at a length of 75bp. All libraries were prepared using a single lot of reagents and equipment and were processed by the same technical staff. Samples were processed in two runs with technical and sample duplicates with negligible batch differences. The data quality check was conducted on an Illumina SAV. Demultiplexing was conducted using the Illumina Bcl2fastq2 program (version 2.17). DESeq2 (version 1.18.1) was applied to produce normalized counts and the data were log2-transformed. RNA-Seq counts are available through NCBI’s Gene Expression Omnibus (GEO) GSE217352.

Transcriptomics data on human whole blood from CD patients who were refractory to anti-tumor necrosis factor-α treatment and who participated in the CERTIFI (7, 8) study were downloaded (Affymetrix HT HG-U133+ PM Array Plate, GSE100833). The data processing methods used were the same as those previously described (9).

### Statistical analysis

RNA-Seq data analysis and data mining were conducted using the BRB array tools (brb.nci.nih.gov/BRB-ArrayTools, version 4.6.1) and the R program (www.r-project.org). Class prediction analysis used Bayesian covariate predictor, diagonal linear discriminant analysis, k-nearest neighbor (using k = 1 and k = 3), nearest centroid, support vector machines, and non-negative matrix factorization multivariate classification methods, based upon a minimum *p*-value of 0.001. A bootstrap cross-validation randomly resampling method was used to compute the misclassification rate. The false discovery rate to control for multiple hypothesis testing was calculated using the Benjamini–Hochberg method. Cluster analysis was conducted using BRB array tools and Cluster 3.0 with Java Treeview. The xCell algorithm and webtool was applied for the T-cell deconvolution of cell-type specific abundance (10). The gene set variation analysis (GSVA) method (11) was used to calculate single-sample gene set enrichment. To derive a single-value continuous metric of gene enrichment capable of differentiating between the CD-PBmu and CD-PBT subtypes, GSVA was conducted using the 42-gene biomarker panel as the gene set. Tests for statistical significance were carried out using JMP Statistical Software (Cary, NC). Data were assessed for normality using the Shapiro–Wilk test. If data were normal, a two-tailed, unpaired Student’s *t*-test was used. For non-normal data, the Wilcoxon or Kolmogorov–Smirnov tests were used. A univariate model was fitted with CD subtypes for demographic and clinical data. There was no statistical significance among any demographic or clinical attributes when comparing the CD-PBmu and CD-PBT subtypes, and multivariate analysis was not conducted. Analysis for identifying the peripheral transcriptional signal alteration after surgery was conducted by comparing the expression of pre- and post-surgery paired samples for individual patients. Similarly, potential CD-PBmu kinase targets were identified by comparing the paired samples taken at the time of surgery for enhanced kinase expression with the postoperative decreased expression selective for the CD-PBmu subtype, but not the CD-PBT subtype.

### Validation of the CD-PBmu signature

Data pertaining to the whole-blood gene expression of CD patients who had participated in the CERTIFI study (7, 8) and who were refractory to anti-TNFα therapy were downloaded (accession number GSE100833). The CD activity index scores for patients in this study were between 220 and 450, and patients had a median disease duration of 11 years. The expression data for validation were the baseline values for patients in whom anti-TNF therapy had been unsuccessful and who had not yet undergone drug treatment (with ustekinumab). The metadata regarding patients’ response to therapy were not publicly available. Hierarchical clustering using the gene signature that defined the CD-PBmu subtype was carried out. The mean percentage of correct cluster classification was determined using the Bayesian covariate predictor, diagonal linear discriminant analysis, k-nearest neighbor (using k = 1 and k = 3), nearest centroid, support vector machines, and non-negative matrix factorization, and a bootstrap cross-validation prediction error of < 0.01 based on 100 bootstrap samples. Cell-type enrichment was determined using xCell (10).

### Pathway analysis and tissue co-expression similarity

Qiagen Ingenuity Pathway Analysis and Ingenuity analysis match (IPA, Qiagen Redwood City; www.qiagen.com/ingenuity) and Enrichr (12, 13) (http://amp.pharm.mssm.edu/Enrichr/) or BRB array tools for GO and KEGG pathway enrichment analysis were used to analyze the pathway enrichment of differentially expressed genes.

The ARCHS4 (14) (https://amp.pharm.mssm.edu/archs4/) database tool was used to identify tissue signature similarity in co-expression. CD-PBmu signature genes (restricted to HGNC-approved symbols) with increased differential expression (≥ 2) and *t* values between 3.5 and 7 (*n* = 193) served as the input. GEO studies with tissue co-expression similarity were downloaded. The 42-gene biomarker classifier was developed by the sequential deletion of individual genes as input for ARCHS4 analysis and by maintaining the GEO mucosal signature for co-expression similarity. Transcriptome-wide association (TWAS) (15) (http://twas-hub.org/genes/) and pleiotropic disease and trait associations were determined using phenome-wide (PheWAS) (16) (https://phewascatalog.org/) tools.

### TRS calculation

We used the methods described in Marigorta et al. (17) to calculate the genetic TRS. Of the 232 known IBD loci, 122 were either cis-eQTLs or in a strong linkage disequilibrium (r^2^ > 0.8), with at least one cis-eQTL in peripheral blood. This corresponds to 163 (157 unique) corresponding eGenes, i.e., ~1.3 candidate genes per SNP. We determined that 142 out of 157 eGenes were present in 17% of the CD-PBmu and PBT samples. All 142 eGenes had cis-eQTLs in previously defined regions (18, 19). Transcript abundance was standardized and polarized according to the direction of risk, as noted previously (17–19). The transcript abundances in cases in which low expression was associated with risk were flipped. The summation of all eGenes provided the TRS, which was further standardized.

### Serological antibody responses

Sera were analyzed for the expression of anti-glycan antibodies to *Saccharomyces cerevisiae* (ASCA), antibodies to the outer-membrane porin C of *Escherichia coli* (OmpC), a *Pseudomonas fluorescens*-associated sequence (I2), antibodies against the flagellin CBir1 (anti-CBir1), and anti-neutrophil cytoplasmic antibodies (ANCA) in a blinded fashion by ELISA as previously described (20). Quartile sum scores were generated as previously described and did not include ANCA (20, 21).

## Results

### A treatment-resistant CD patient population is characterized by mucosal-cell-like T cells circulating in the periphery

**Figure 1:**
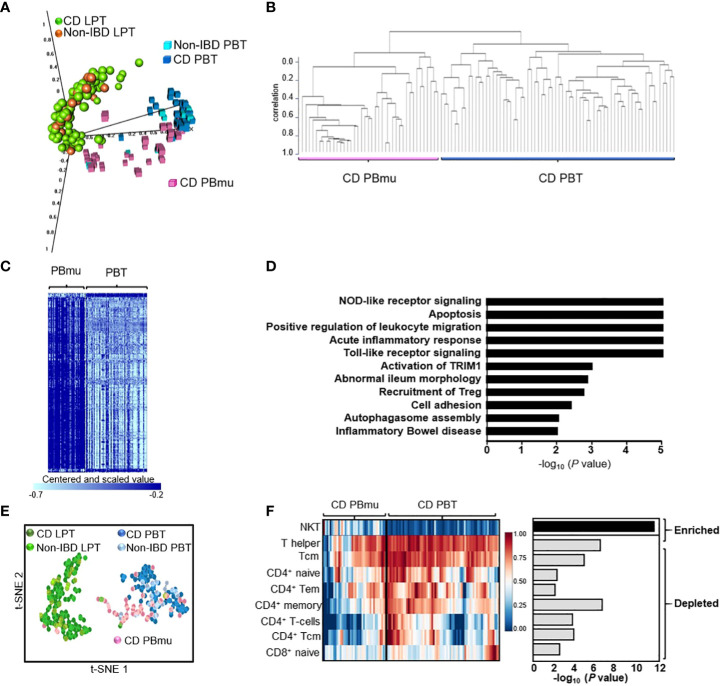
The identification of a novel CD patient subtype with a circulating mucosal cell-like gene expression profile. **(A)** Principal component analysis (PCA) of CD3^+^ T-cell gene expression from the lamina propria or periphery isolated from CD patients or non-IBD individuals. **(B)** Unsupervised hierarchical clustering defining two CD peripheral expression subtypes, CD-PBmu and CD-PBT. **(C)** Heat map of 1,566 differentially expressed genes between the CD-PBmu and CD-PBT subtypes (*p*-value < 0.001, FDR <0.002, and fold change ≥ 2). **(D)** Pathway analysis of differentially expressed genes in the CD-PBmu subtype. **(E)** t-SNE plot of deconvoluted CD3^+^ immune cell enrichment scores. **(F)** Heat map and *p*-values of altered T-cell subset abundance in the CD-PBmu vs. CD-PBT subtypes (Mann–Whitney test).

We sought to identify the molecular pathways underlying T-cell transcriptomic signatures in treatment-resistant CD patients whose disease required surgical intervention. Purified CD3^+^ T cells were isolated from matched paired samples from the peripheral blood and mucosa of 100 CD patients and 17 non-IBD control subjects taken at the time of surgery. Principal component analysis (PCA) of gene expression enabled us to distinguish between the lamina propria mucosa-derived (mucosal; CD LPT) T cells and those in the periphery (**Figure 1A**). Among mucosal T cells, the expression profiles of CD patients and non-IBD control subjects were interspersed. In contrast, among peripheral T cells, we observed two distinct CD transcriptomic signatures. One expression signature, designated CD-PBT (63%), clustered tightly with non-IBD subjects. A second peripheral expression signature was shifted towards the mucosal T-cell signature and was designated CD-PBmu(cosal) (37%) (**Figures 1A, B**). The subtype classification (≥ 90%) was confirmed using multiple statistical techniques (**Supplemental Table 1A**). We identified 1,566 genes with ≥ twofold differential expression among the CD-PBmu and CD-PBT subtypes (*p* < 0.001, FDR < 0.002) (**Figure 1C** and **Supplemental Table 2**). Less than 5% of these genes were differentially expressed when comparing CD-PBmu with CD-LPT cells or with non-IBD LPT cells supporting a CD-PBmu mucosal-like signature (**Supplemental Figure 1**). Among these genes, > 90% were overexpressed in the CD-PBmu subtype. Pathway analysis indicated that in the CD-PBmu subtype differentially expressed genes (DEG) were enriched in the pathways associated with T-cell activation, leukocyte adhesion/migration, and integrin-binding features. These mucosal cell-like features suggest that the CD-PBmu subtype represents recent mucosal emigrants (**Figure 1D**).

We assessed whether or not the transcriptomic signature stratifying the CD-PBmu and CD-PBT subtypes was associated with clinically relevant disease markers that reflected a greater mucosal inflammatory disease burden prior to surgery. To minimize variability in clinical assessment, patients were characterized, and surgical samples collected from resections carried out by a single surgical provider. No significant differences were noted between the demographics of the CD-PBmu and CD-PBT patient populations (**Supplemental Figure 2** and **Supplemental Table 3**). Moreover, there were no significant associations in the key indicators of disease burden, including disease location or behavior, length or location of intestinal resection, and pre-operative medications. Interestingly, only one individual classified as having the CD-PBmu subtype was a current smoker, a characteristic that is commonly associated with severe disease. In addition, both a pre-operative disease severity score based on a weighted disease index (3) and an in-house gross surgical pathological severity score based on the depth and extent of inflammation in the resected segment were calculated. These severity scores also failed to stratify the peripheral CD-PBmu and CD-PBT subtypes, therefore validating the experimental design in that patients with severe disease displayed a transcriptomic signature that was not merely reflective of a global enhanced inflammation in the CD-PBmu subtype.

### The imputed composition of peripheral T-cell subsets is altered in the CD-PBmu subtype

CD3^+^ T cells are a heterogeneous population with a mosaic of naive, activated, memory and effector T-cell traits defined by their cell surface markers and immune response. The abundance of individual subsets can be quantified from RNA sequencing data using bioinformatic approaches. We hypothesized that the distinct transcriptomic signatures observed between the CD-PBmu and CD-PBT subtypes may result from an underlying alteration in peripheral T-cell subset composition. Individual immune cell enrichment scores were calculated and a t-SNE analysis was carried out. As seen in **Figure 1E**, the t-SNE cell signature enrichment plot mimics that observed for the gene expression (**Figure 1A**) with distinct clustering of the CD-PBmu and CD-PBT subtypes. The comparison of the CD-PBmu subtype with the CD-PBT subtype demonstrated the inferred enrichment of natural killer T (NKT) cells and the depletion of T helper 1 (TH1) and CD4^+^ and CD8^+^ memory and naive cell subsets (**Figure 1F**). The enrichment scores do not infer percentage comparison across cell types, i.e., the enrichment of NKT cells does not necessarily correlate with the depletion of CD4^+^/CD8^+^ cells. Indeed, no significant correlation was noted between the NKT and CD4^+^/CD8^+^ T-cell subset enrichment scores (**Supplemental Figure 3**). To further confirm the deconvolution analysis, the CD-PBmu and CD-PBT subtypes were compared using the ingenuity analysis match metadata evaluator method. We again identified differential gene expression and upstream regulatory pathways that had previously been identified when comparing NKT cell with CD4^+^ T-cell subsets (**Supplemental Table 4**), supporting the findings we obtained by deconvolution of the CD3^+^ T-cell composition.

### The CD-PBmu transcriptomic signature was validated in an independent cohort

**Figure 2:**
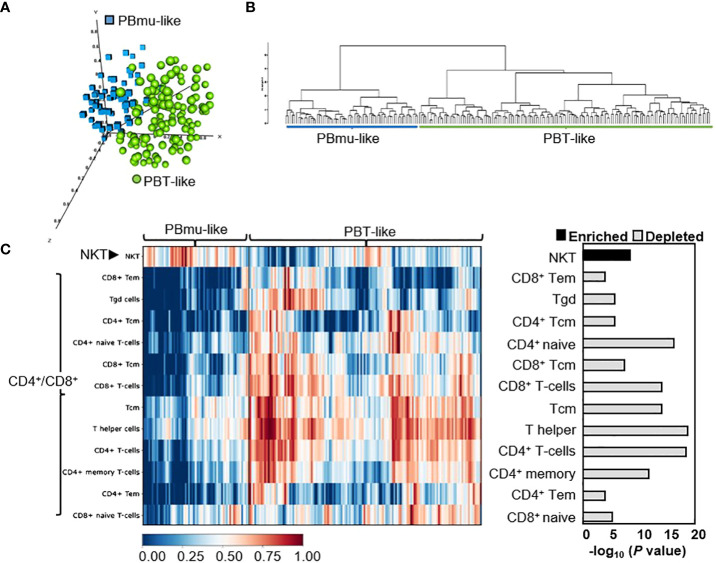
CD-PBmu expression signature stratified CD patients in whom anti-TNF therapy was unsuccessful. **(A, B)** The genes defining the CD-PBmu vs. CD-PBT subtypes (**Figure 1C**) were used to identify similar subtypes from an independent CD cohort of patients in which anti-TNF therapy was unsuccessful. **(A)** Principal component analysis (PCA) and **(B)** hierarchical clustering of expression data identified two CD patient subtypes. **(C)** Heat map based on cellular enrichment scores produced using the xCell bioinformatics tool. The enrichment of NKT cell and depletion of CD4^+^/CD8^+^ T-cell subsets were associated with the samples classified as CD-PBmu-like subtype.

We tested the reproducibility of the CD-PBmu transcriptomic signature to identify CD patient subtypes using an independent treatment-resistant cohort and dataset: gene expression in the whole blood isolated from CD patients in whom anti-TNFα therapy had been unsuccessful. These patients exhibited moderate to severe disease, with CD activity index scores of between 220 and 450, and the median disease duration in this patient group was 11 years. Patients with strictures that could require surgery or those having undergone bowel resection within the past 6 months were excluded. The expression data collected for validation were taken from CD patients at baseline in whom anti-TNF therapy had been unsuccessful and who had not undergone drug treatment. Hierarchical clustering using the gene set which had defined the CD-PBmu subtype (1,566 transcripts) identified two distinct PBmu- and PBT-like clusters (**Figures 2A, B**). PCA and differential gene expression distinguished between these groups, with approximately 31% of patients displaying a CD-PBmu-like expression pattern and an average classification performance of 92% (**Supplemental Table 1B**). Moreover, cell-type enrichment analysis revealed that there was a similar inherent imbalance of T-cell subsets with the enrichment of NKT cells and depletion of CD4^+^/CD8^+^ subsets associated with the PBmu-like classification (**Figure 2C**). The imbalance in T-cell subset composition was distinct for the CD-PBmu signature and was not observed when applying a random probe-gene set for clustering analysis (**Supplemental Figure 4**). The clinical metadata of patient response to ustekinumab were not publicly available and thus we were unable to establish whether or not the PBmu-like signature corresponded with a distinctive clinical outcome.

### The peripheral T-cell subset composition in the CD-PBmu subtype is associated with distinct clinical and serological characteristics of disease severity

**Figure 3:**
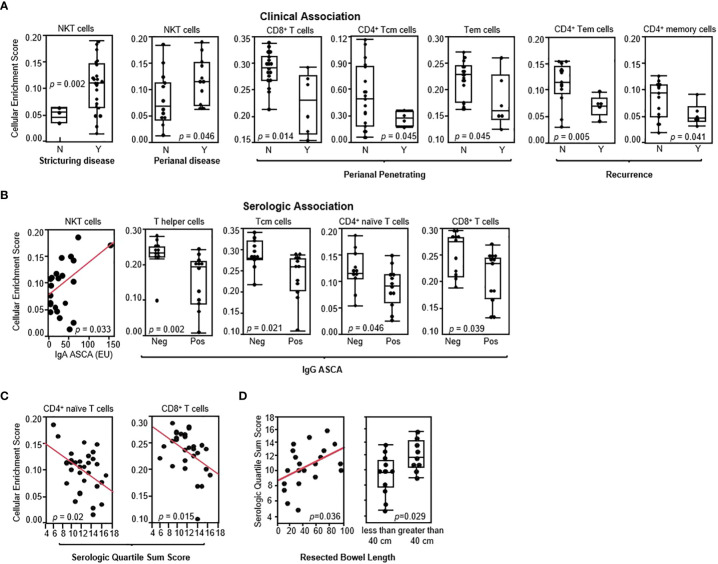
CD-PBmu-altered T-cell subset composition was associated with the clinical and serological parameters of complicated disease. **(A)** The association of NKT cell enrichment with stricturing disease and perianal disease and CD4^+^/CD8^+^ T-cell subset depletion in the CD-PBmu subtype with perianal penetrating disease and postoperative endoscopic recurrence (N= Rutgeerts score 0–1; Y=2–4) **(B)** Association of NKT cell enrichment and CD4^+^/CD8^+^ T cell subset depletion in the CD-PBmu subtype with ASCA seropositivity. **(C)** Inverse correlation of serological quartile sum scores in CD-PBmu with CD4^+^/CD8^+^ T-cell subsets depletion. **(D)** Association of serological quartile sum scores in the CD-PBmu subtype with increased length of bowel resection.

The impact of altered gene expression and T-cell subset composition on the clinical characteristics of disease activity was assessed. In the CD-PBmu (**Figure 3A**) subtype, but not in the CD-PBT subtype (**Supplemental Figure S5**), NKT cell enrichment scores were associated with stricturing disease at the time of surgery (**Figure 3A**). Interestingly, although only a small patient cohort was studied, we were able to observe that those patients with the CD-PBmu subtype were significantly more likely to develop stricturing disease than those with the CD-PBT subtype (Cochran Armitage trend test, *p* = 0.033). The presence of perianal disease at the time of surgery was also associated with enrichment in NKT cells (**Figure 3A**). Furthermore, the depletion of CD4^+^ and CD8^+^ T-cell subsets observed in both the CD-PBmu and CD-PBT subtypes was associated with perianal penetrating disease and the postoperative endoscopic recurrence of disease (the average interval for postoperative evaluation in both the CD-PBmu and CD-PBT subtypes was 10 months) (**Figure 3A**). In CD patients, serologic responses to commensal bacteria and auto-antigens such as ASCA, OmpC, I2, and anti-CBir1 have been associated with more severe clinical disease phenotypes and the risk of complications (20, 22–24). In particular, a high antibody response toward multiple microbial antigens is predictive of aggressive disease and risk for surgery (20, 23). In the CD-PBmu subtype, but not in the CD-PBT subtype, the NKT cell enrichment scores correlated with increased ASCA seropositivity levels (**Figure 3B**). Conversely, the depletion of CD4^+^/CD8^+^ T-cell subsets was associated with ASCA positivity. Moreover, in the CD-PBmu subtype, but not in the CD-PBT subtype, the depletion of CD4^+^ naive and CD8^+^ T cells was associated with enhanced serological quartile sum scores of response (**Figure 3C**), and enhanced serological quartile sum scores of response to multiple microbial antigens in the CD-PBmu subtype were associated with an increased resected intestine length (**Figure 3D**). These findings suggest that an altered T cell subset composition characterized by the CD-PBmu subtype may help in the substratification of disease within a patient population that is resistant to therapeutic intervention.

### CD-PBmu vs. CD-PBT is associated with an elevated genetic transcriptional risk score

**Figure 4:**
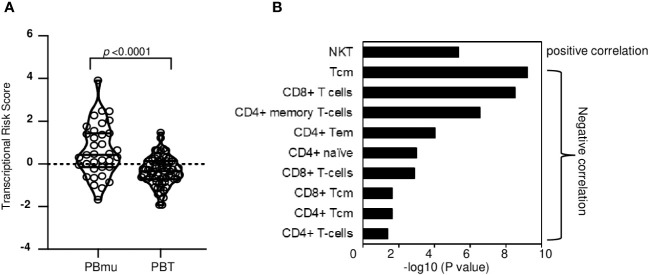
Genetic association of TRS with CD-PBmu signature **(A)** The CD-PBm subtype was associated with higher TRSs than the CD-PBT subtype. **(B)** Association of NKT cell enrichment and CD4^+^/CD8^+^ T-cell subset depletion with TRS.

To evaluate the extent to which genetic variation contributes to the transcriptomic profile and biologic processes that ultimately define the CD-PBmu subtype, genetic TRSs were generated, integrating IBD GWAS-associated variants and the expression of quantitative trait loci (eQTL) with transcriptional data. A higher genetic TRS has been reported to be associated with disease severity and can better predict disease progression compared with genetic risk scores (17). The CD-PBmu subtype was associated with a significantly higher genetic TRS than the CD-PBT subtype (*p* < 0.001) (**Figure 4A**). Moreover, the TRS demonstrated a positive correlation with NKT and a negative correlation with CD4^+^/CD8^+^ memory and naive cell subset enrichment scores (**Figure 4B**). In terms of genetic risk scores, there was no significant association seen in the CD-PBmu subtype vs. the CD-PBT subtype.

### The CD-PBmu subtype transcriptomic signature reverted to that observed for the CD-PBT subtype following surgery

**Figure 5:**
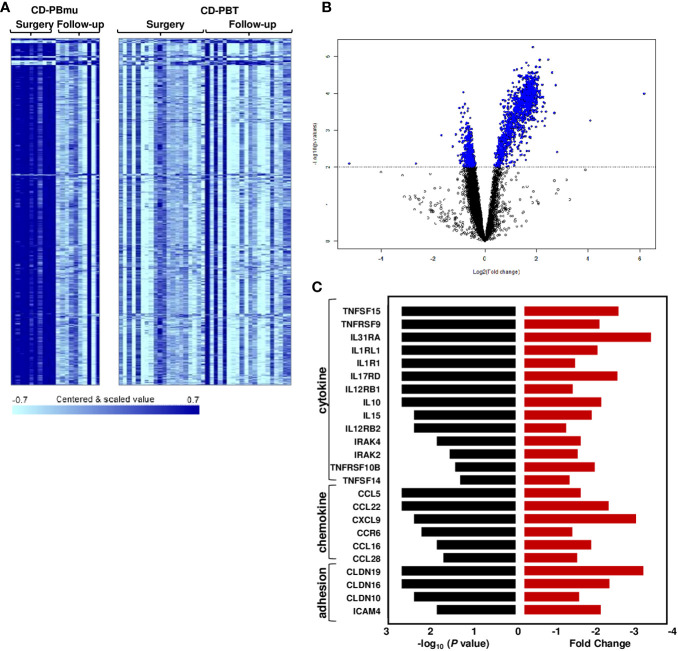
Postoperative changes in CD-PBmu gene expression profile. **(A)** Heat map and **(B)** volcano plot of 877 genes differentially expressed in the CD-PBmu subtype at the time of surgery vs. post-operatively (*p*-value < 0.001, FDR < 0.01). **(C)** Attenuation of proinflammatory cytokine, chemokine, and adhesion molecule expression in the CD-PBmu subtype following surgery. The black bars represent *p*-values and the red bars represent the corresponding fold change.

Additional samples were collected from 30 CD patients 3–13 months post-surgery to assess the stability of the transcriptomic profiles. In patients classified as having the CD-PBmu subtype at surgery, there was a significant alteration in gene expression at the subsequent collection following surgery (877 genes, *p* < 0.001, FDR < 0.013). Notably, the overexpressed transcriptomic signature that had defined the CD-PBmu subtype at the time of surgery was no longer present after surgery (**Figures 5A, B**). Similarly, there was a downregulation of pro-inflammatory cytokine, chemokine, and adhesion molecule expression following surgery (**Figure 5C**). In addition, there was a significant decrease in the level of NKT cells (*p* = 0.026) and an increase in TH1 cells (p = 5e-03) in the CD-PBmu patient population following surgery. As seen in **Supplemental Figure 6**, the gene expression of the CD-PBmu subtype reverts after surgery to that observed for the CD-PBT patients and non-IBD subjects at the time of surgery, demonstrating a high correlation in expression between CD-PBmu subtype samples following surgery and pre- and post-surgery CD-PBT subtype samples. A separate independent CD cohort assessing the attenuation of the CD-PBmu profile (*n* = 19) following surgery validated these findings (**Supplemental Figure 7**). As seen in the PCA and heat map plots there is a clear distinction in expression between the CD-PBmu and CD-PBT subtypes at the time of surgery (**Supplemental Figures 7A–C**). Furthermore, the genes defining the pre- and post-surgery CD-PBmu samples in the initial cohort were validated and demonstrated an alteration in gene expression post-surgery exclusively in the CD-PBmu subtype (PCA and heat map analysis, **Supplemental Figures 6D–F**). However, no alteration in gene expression was detected in the CD-PBT subtype post-surgery.

### The upregulated transcriptomic signature of the CD-PBmu subtype is similar to that of ileal biopsy samples from treatment-naive pediatric patients with CD

We utilized the ARCHS4 tool to compare the similarity of CD-PBmu transcriptomic signatures across multiple independent RNAseq studies (26,876 samples) to determine in turn the relationship between gene expression and disease. A panel of the first genes with a ≥ two-fold upregulated DGE (t value 3.5–7, *n* = 193) was used for analysis and the samples identified using the ARCHS4 tool as matching the CD-PBmu input signature were downloaded. As seen in **Supplemental Figure 8** and **Supplemental Table 5**, the peripheral CD-PBmu signature highly correlated with the expression of ileal biopsy samples from inception studies on treatment-naive pediatric patients with CD. The similarity of the CD-PBmu signature with ileal biopsy samples substantiates the idea that circulating CD-PBmu peripheral T cells have a mucosal origin.

**Figure 6:**
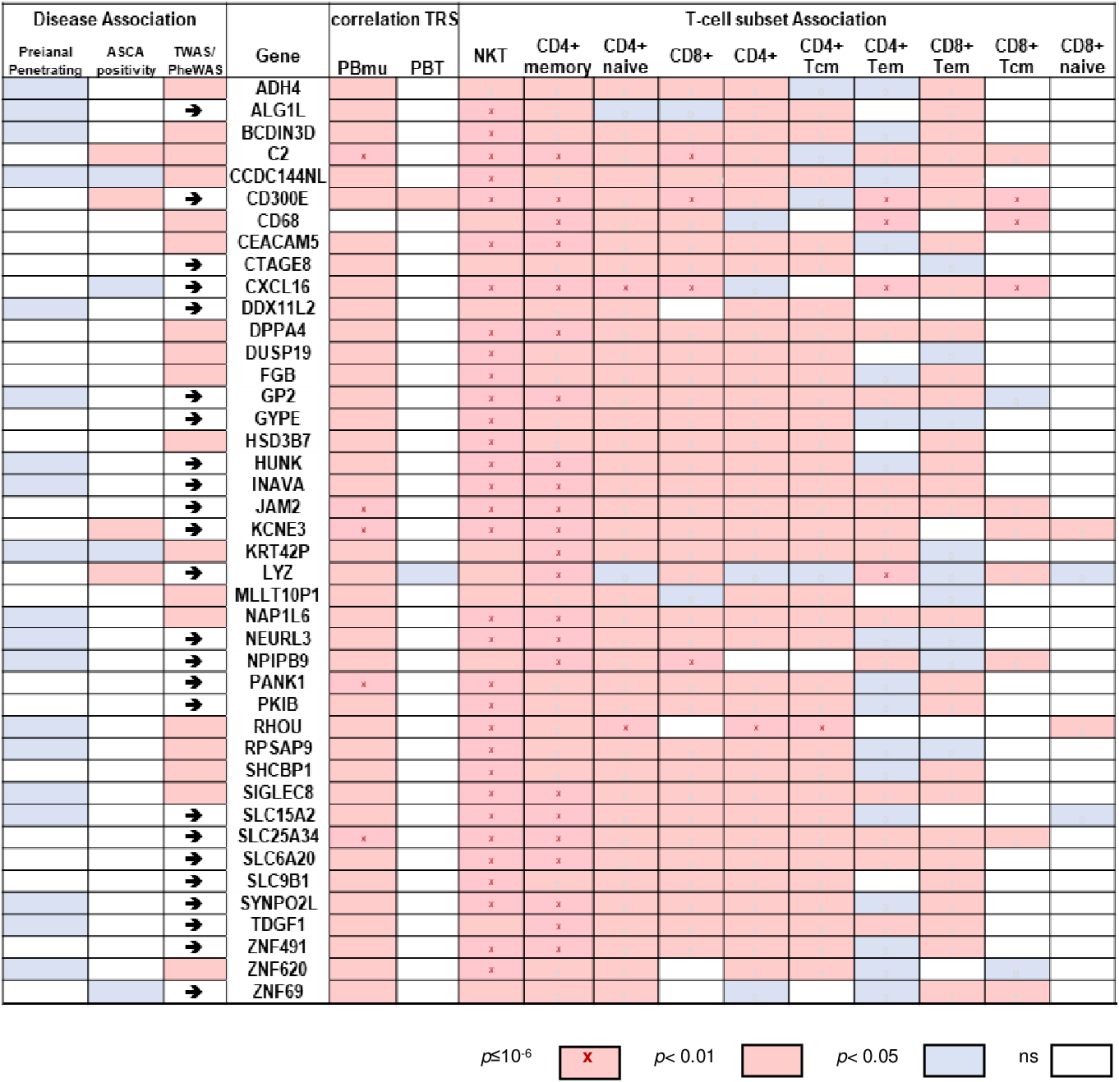
Expression of 42-gene biomarker panel correlated with CD-PBmu genetic TRS and altered T-cell subset composition. Heat map of correlation values of gene expression vs. TRS and enrichment scores for biomarker panel (right panel) and association with perianal penetrating disease and ASCA sero-positivity (left panel). The arrows highlight a reported TWAS IBD association.

A 42-gene biomarker classifier was developed, which sustained the co-expression similarity of the peripheral CD-PBmu and CD-PBT signatures and in which treatment-naive pediatric patient mucosal samples functioned as a discriminator. All 42 genes displayed a significant positive correlation with the NKT cell enrichment scores with the majority (33 out of 42) associated with a *p*-value of < 1E-06 (**Figure 6**). Conversely, there was a negative correlation (**Figure 6** and **Supplemental Figure 9**) between the gene panel expression and the CD4^+^/CD8^+^ T-cell enrichment scores. Similarly, the results of a gene set variation analysis (GSVA) conducted to derive a single-value continuous metric of the 42-gene biomarker expression across all samples demonstrated significant correlation with T-cell subset enrichment scores (**Supplemental Figure 10**). The biomarker classifier maintained the CD-PBmu and CD-PBT classification (82% accuracy, non-negative matrix factorization clustering). The 42-gene biomarker panel was assessed for genetic association. The expression of 41 out of 42 biomarker genes was significantly correlated (*p* < 0.01) with CD-PBmu genetic TRSs, whereas only 1 out of 42 genes reached significance (*p* < 0.05) for the CD-PBT subtype. Moreover, the 42-gene panel overlapped with TWAS signals predicted for associations with IBD and clinical association to perianal penetrating disease and ASCA seropositivity (79% of panel) (**Figure 6**).

### Identification of potential protein kinase signaling pathways regulating expression of the CD-PBmu transcriptomic signature

**Figure 7:**
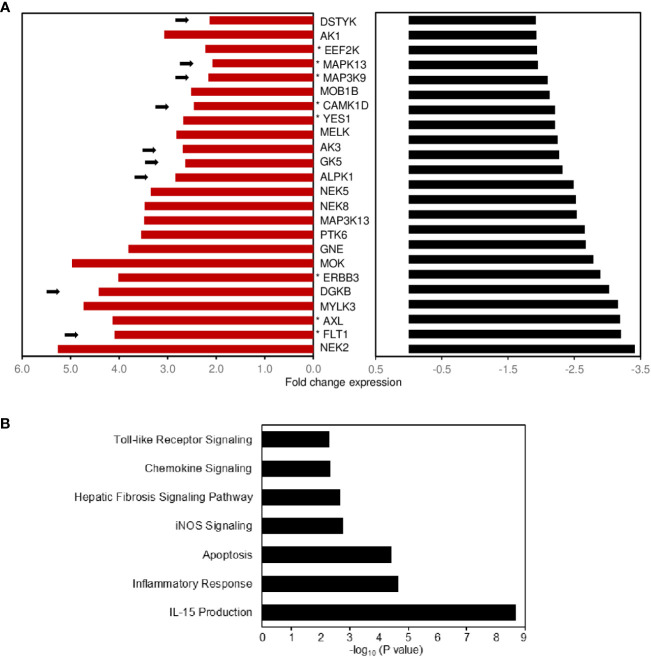
Potential protein kinase signaling pathways regulating expression of the CD-PBmu expression signature. **(A)** Bar plot shows the fold enhancement of kinase expression when comparing the CD-PBmu subtype with the CD-PBT subtype prior to surgery (red bars) and selective decrease post-operatively for the CD-PBmu subtype (black bars). The arrows highlight a reported TWAS IBD association and the asterisk therapeutic kinase inhibitors currently in use or in clinical trials. **(B)** Pathway analysis of differentially expressed kinase genes in the CD-PBmu subtype prior to vs. post-surgery.

Protein kinases are known mediators of chronic inflammation, activating the signaling pathways involved in cytokine/chemokine secretion, cellular activation, adhesion, and migration. Protein kinases also play a significant role in mediating the pathogenesis of IBD. There is great interest in understanding how kinases are regulated by protein–protein interactions with a view to identifying new therapeutic targets for development. To discover which candidate kinases potentially regulate CD-PBmu DGE, we first identified the kinases that displayed a co-occurrence of selective enhanced expression prior to surgery and an associated selective decrease post-operatively in the CD-PBmu and CD-PBT subtypes (**Figure 7A**). For CD-PBmu, 24 kinases displayed increased expression prior to surgery and post-surgical attenuation (˜twofold) selectively for CD-PBmu. Pathway analysis indicated that these kinases were associated with IL-15 production, toll-like receptor and chemokine signaling, inflammatory response, and apoptosis (**Figure 7B**). Furthermore, 38% of these kinases (9 out of 24) were associated with an IBD TWAS/GWAS signal, and 29% were targets of currently approved kinase inhibitors (7 out of 24), which might conceivably be repurposed for the management of severe CD.

## Discussion

Despite significant advances brought about by biologic therapies for inflammatory bowel disease, many individuals with CD experience persistent active disease, elevated rates of recurrence, and require surgical intervention. CD is associated with a significant burden of healthcare costs and reduced quality of life. Currently, there is no reliable molecular diagnostic approach that can be used to predict a lack of therapeutic response or postoperative recurrence in patients. This study focused on a CD patient population with severe disease, with the goal of identifying the molecular pathways underlying the clinical disease course. We characterized a circulating peripheral T-cell transcriptomic signature that substratified these patients into two distinct molecular subtypes, which we have termed the CD-PBmu and CD-PBT subtypes. Patients exhibiting a CD-PBT transcriptomic signature clustered tightly with non-IBD subjects. Patients classified as having the CD-PBmu subtype displayed a transcriptomic signature that approached a mucosal T-cell profile that mirrored an alteration in the inferred T-cell subset composition and correlated with a distinct subset of clinical features associated with complicated/aggressive disease. The CD-PBmu expression signature was associated with an elevated genetic TRS, supporting the existence an underlying pathogenetic relationship. Moreover, it was only among CD-PBmu patients that circulating peripheral T cells exhibited the marked downregulation of pro-inflammatory and adhesion molecule expression following surgical resection of the inflamed bowel tissue. These findings provide evidence for the novel classification of biologically distinct subtypes in CD patients with severe medically refractory disease based upon their circulating peripheral T-cell transcriptomic signatures.

The high clinical heterogeneity and genetic complexity of CD has revealed that the underlying biological pathways driving this disease differ among patients. Genetic, molecular, immunologic, and microbiome studies provide evidence that this complexity reflects both the spectral and modal combinations of these traits (5, 18, 19, 25–27), including the potentially targetable causal pathways. Thus, the development of targeted therapeutics early in the course of disease requires the use of biomarkers that are qualified in defining such subgroups.

The significance of the unique CD-PBmu transcriptomic signature is twofold. It has the prognostic potential, which, together with a genetic component, allow it to identify, in a minimally invasive manner, a subset of CD patients that is likely to develop severe disease, which might be averted through early initiation with individualized therapy. Second, the transcriptomic signature has potential as a first step in the development of a companion diagnostic to identify and predict patient response to a particular drug or therapeutic pathway, for example kinase targets. It is important to put these findings within the context of other biomarker studies.

The mucosal gene expression in non-inflamed colon tissue from CD adults undergoing surgery, and to a lesser extent from treatment-naive pediatric CD patients, has been classified into a colon-like profile (associated with rectal disease) and an ileum-like profile (associated with the need for postoperative biological therapy) (4). Peripheral T cells isolated from CD-PBmu patients showed DGE of both ileal and colonic signature genes compared with both CD-PBT and non-IBD subjects. No subtype DGE was seen in the mucosal compartment.

A separate study examined peripheral CD8^+^ T-cell gene expression in treatment-naive IBD patients at the time of diagnosis and a whole-blood gene expression panel was designed to stratify patients and predict the risk for a chronic relapsing vs. mild disease course (28, 29). In the context of our patient population with refractory disease, in which aggressive treatment had been unsuccessful and which required surgical intervention, the whole-blood panel was unable to identify the distinct patient subtypes. These findings indicate that the tissue source, and also response to therapy and disease behavior, are critical factors to be considered when defining biologically distinct CD subtypes. Notably, even within the periphery, the pathways defining patients at risk for more complicated disease may not be straightforward. The molecular classification presented here identifying two clinically relevant CD subtypes is unique in that it provides evidence for heterogeneity in a patient population in which therapeutic treatment escalation was unsuccessful, and the patients of which had similar pre-operative severity scores and required surgical resection. It is interesting to note that although only a small number of patients were recruited, the peripheral gene biomarker substratified the inception treatment-naive pediatric CD ileal cohort at diagnosis and projected the likelihood for disease progression to stricturing and/or penetrating disease. Studies that aim to confirm the presence and predictive value of the peripheral CD-PBmu signature early on in a pediatric cohort have been planned. The presence of the CD-PBmu gene signature in the whole blood of a subset of CD patients in whom anti-TNF therapy was unsuccessful, and the TRS genetic association of our CD-PBmu biomarker panel, indicate a potential clinical application to facilitate patient stratification and more effective treatment prior to surgical resection.

The balance of T-cell trafficking from the periphery into the gut and subsequent recycling of activated T cells back to the periphery is tightly regulated and is essential for maintaining immune gut homoeostasis. Uncontrolled chronic intestinal inflammation in CD is characterized by the infiltration of circulating activated proinflammatory T cells in the mucosa. CD4^+^ T-cell infiltration in the intestinal tissue of IBD patients is a key feature of chronic intestinal inflammation with enhanced accumulation in active disease (30, 31). An imbalance in the mucosal NKT cell population has also been reported in CD patients with severe disease (32). A number of studies have also identified an imbalance in other mucosal T-cell subsets, including T regulatory cells (Tregs) and central memory T cells (Tcms) associated with disease activity (5, 33, 34). However, the prognostic utility of such findings is limited in that mucosal sampling requires invasive procedures and often the site of disease is difficult to access. Alterations in circulating T- and B-cell activation markers have been reported during disease flare and remission in CD and ulcerative colitis (UC) patients (35). Interestingly, a recent study identified enrichment in an unconventional NKT invariant cell population in patients with CD vs. healthy subjects (36). However, the differential gene expression which characterized this cell population failed to substratify our patients with refractory disease who were undergoing surgical intervention. An emerging body of evidence suggests that “gut-tropic” circulating lymphocytes play an important role. It is therefore of particular significance that we have identified a subset of CD patients with a circulating blood transcriptomic signature associated with a mucosal cell-like expression profile that reverts following the surgical removal of the inflamed tissue. One plausible explanation might be that this patient population exhibits a distinct inflammation-mediated spilling of activated mucosal cells into the periphery. The expression of both CCR9 and CCR6 gut homing chemokine receptors was elevated in the peripheral blood of patients with the CD-PBmu vs. that of patients with the CD-PBT subtype. In fact, the balance of the T-cell composition ratio in matched paired samples between the periphery and mucosa was skewed in the CD-PBmu patient subtype, with a more pronounced increase in the peripheral NKT signature and an associated pronounced decrease in the level of mucosal T cells compared with those with the CD-PBT subtype. Conversely, an inverse skewed balance between the periphery and mucosa was seen for the CD4^+^ memory T-cell signature. Although our findings are based upon imputed CD-PBmu cell subset imbalance, they provide a solid foundation for the further evaluation of alterations in T-cell subsets in future studies. These findings suggest that the dysregulation of circulating intestinal‐homing lymphocytes within the CD-PBmu subtype may underlie the molecular pathways mediating uncontrolled intestinal inflammation within this patient population.

Kinase dysregulation has been demonstrated as an underlying mechanism involved in the pathogenesis of IBD (37). Kinase inhibitor drug discovery is therefore a focus in the search for new therapeutic options. The CD-PBmu transcriptomic signature provides independent evidence of plausible kinase targets that are particularly relevant to severe disease and may aid in the prioritization of new drug development and in guiding decisions as to which patients may benefit most from these targeted strategies. Therapeutic inhibitors for seven of the identified kinase targets, which may enable accelerated drug repositioning, are currently in use or in clinical trials. A number of the potential CD-PBmu kinase targets are associated with the activation of the mitogen-activated protein (MAP) signaling pathway. Similarly, many are intertwined and have been associated with IBD. IL-15, for example, enhances T-cell activation and is elevated in inflamed IBD mucosa (38), whereas a decrease in IL-15 is associated with a positive response to anti-TNF therapy. Both upstream IL-15 and downstream kinase modulators, i.e., YES1, AXL, and FLT1, were identified as potential CD-PBmu targets. In addition, downstream regulators of IL-15 include members of the Janus kinase (JAK)-signal transducer and activator of transcription (STAT) pathway. Numerous therapeutic agents that target members of this pathway have been developed. In particular, there has been an interest in the potential use of JAK inhibitors in therapeutic interventions for IBD. Tofacitinib, a JAK inhibitor and potent inhibitor of IL-15 signaling, was recently approved for use in treating patients with ulcerative colitis (39). In addition, baricitinib, a JAK inhibitor approved for the treatment of rheumatoid arthritis, targets multiple identified CD-PBmu kinases, suggesting that it could potentially be repurposed as a novel IBD therapeutic. It is interesting to note the association of vascular endothelial growth factor receptor (FLT)1 kinase with the CD-PBmu signature. FLT1 mRNA is increased in active UC (40) and has been identified as a regulator of pulmonary (41), kidney (42), and liver fibrosis (43), and may serve as a potential new drug target for attenuating fibrosis in IBD. To our knowledge, this is the first report to address transcriptomic changes in peripheral T cells in CD patients prior to and following surgery. Transcriptomic changes after surgery were detected selectively in CD patients classified as having the CD-PBmu subtype signature. Moreover, in contrast to serologic inflammatory markers that provide associative rather than causative information, attenuation of proinflammatory cytokines, the expression of chemokines and adhesion molecules after surgical resection likely provides insight into the contributing pathways underlying inflammation in these patients. Recent intriguing evidence suggests that early surgical intervention may in fact improve the disease outcome in a select CD population with ileo-colonic disease (44–47). Considering that post-surgical alteration in gene expression was observed exclusively in the CD-PBmu subtype, the transcriptomic signature, together with the genetic element, might provide insight into the biological underpinnings toward characterization of a patient population who might benefit from early surgical intervention. Future studies are needed as we continue to monitor these patients to determine the role of these transcriptional changes in the ensuing disease course.

This study has strengths in that it is focused on identifying the molecular pathways in an understudied CD patient population with refractory disease in whom therapeutic intervention was unsuccessful; however, it also has some limitations. Although the use of non-IBD subjects as a control group facilitated in the identification of the CD-PBmu subset, the implication of the gene signature and impact upon CD patients with active disease and predicting response to treatment remains to be explored further. In addition, among mucosal T cells, the expression profile of CD patients and non-IBD control subjects were interspersed and indeed there was no differential gene expression when comparing CD vs. non-IBD subjects. This is not the case in the circulating T cells in the periphery. These results were rather unexpected. Uncontrolled chronic intestinal inflammation in disease, whether it is IBD-related or not, is generally characterized by infiltration of circulating activated proinflammatory T cells into the mucosa. It is believed that the balance of T-cell trafficking from the periphery into the gut and subsequent recycling of activated T cells back to the periphery is tightly regulated and essential for maintaining immune gut homoeostasis. In fact, the overlap similarity of the peripheral CD-PBmu signature with the expression from ileal biopsy samples from treatment-naive CD pediatric patients suggests that the signature may have predictive value in determining the disease course. However, the results from this study have yet to be validated and further studies are needed to evaluate the mucosal immune cell contribution over the course of this disease.

In conclusion, we believe that this study will serve as a basis for the characterization of molecular signatures and may assist in providing promising prognostic biomarkers and therapeutic targets for this patient population experiencing the challenges associated with severe CD.

## Data availability statement

The datasets presented in this study can be found in online repositories. The names of the repository/repositories and accession number(s) can be found below: NCBI's Gene Expression Omnibus (GEO), https://www.ncbi.nlm.nih.gov/geo/, accession number GSE217352.

## Ethics statement

The studies involving humans were approved by Institutional Review Board at Cedars-Sinai. The studies were conducted in accordance with the local legislation and institutional requirements. The participants provided their written informed consent to participate in this study.

## Author contributions

RG and ST conceptualized, coordinated, and supervised the study design. PF and EA provided the clinical data. Acquisition, analysis, and interpretation of data: PF, EA, GB, AP, and RG. JB provided important intellectual content. Drafting of the manuscript: RG. Revision of the manuscript: RG, DM, JB, and ST. Funding acquisition: ST and DM. All authors contributed to the article and approved the submitted version.

## Glossary

**Table:**

AKT1	AKT serine/threonine kinase 1
ASCA	anti-Saccharomices cerevisiae antibodies
anti-CBir1	anti-flagellin CBir1 antibody
CD	Crohn’s disease
CDK	cyclin-dependent kinase
CK2	casein kinase 2
CSNK2A1	casein kinase 2 alpha 1
ELISA	enzyme-linked immunosorbent assay
FLT1	vascular endothelial growth factor receptor 1 kinase
GSK3β	glycogen synthase kinase 3 beta
I2	anti-I2 component of *Pseudomonas fluorescens*
IBD	inflammatory bowel diseases
IPA	ingenuity pathway analysis
JAK	Janus kinase
JNK	c-Jun-terminal kinases
LPMC	lamina propria mononuclear cells
MAP	mitogen-activated protein
mTOR	mammalian target of rapamycin
NF−κB	nuclear factor kappa-light-chain-enhancer of activated B cells
OmpC	anti-outer-membrane porin C of *Escherichia coli*
PCA	principal component analysis
PI3K	phosphatidylinositol-3-kinase
PheWAS	phenome-wide association study
RIPK	receptor interacting protein kinase
STAT	signal transducer and activator of transcription
TRS	transcriptional risk scores
TWAS	transcriptome-wide association study
