# Supplementary material for: A blood-based transcriptomic signature stratifies severe Crohn’s disease and defines potentially targetable therapeutic pathways
Source: Front Gastroenterol (Lausanne). 2023 Oct 18;2:1251133. doi: 10.3389/fgstr.2023.1251133 (PMC12952313; doi:10.3389/fgstr.2023.1251133)
Supplement: Supplementary file 1 [file DataSheet_1.pdf]

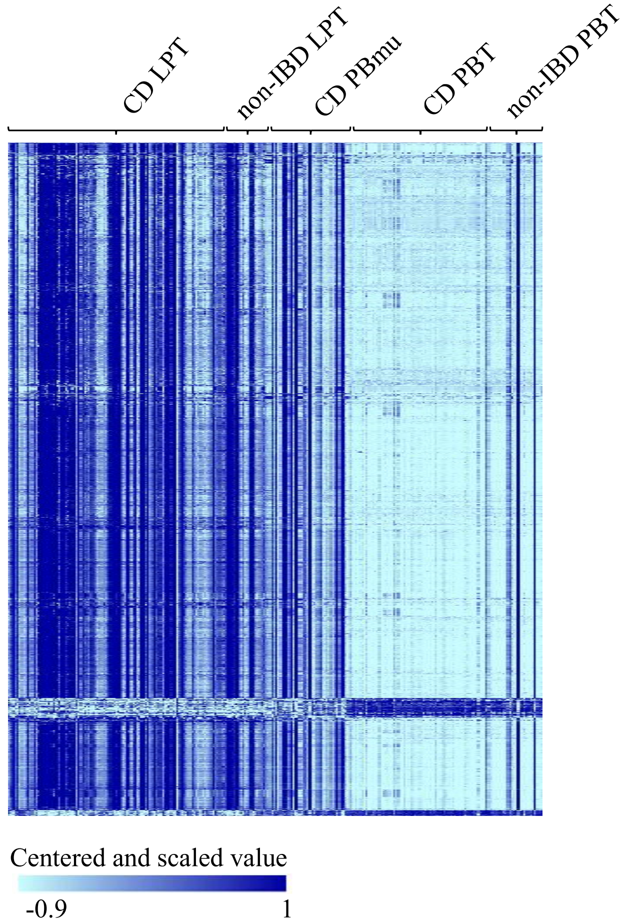

Figure S1: Heatmap of 1566 of CD PBmu and CD PBT subtypes differentially expressed genes across all LPT and PBT samples.

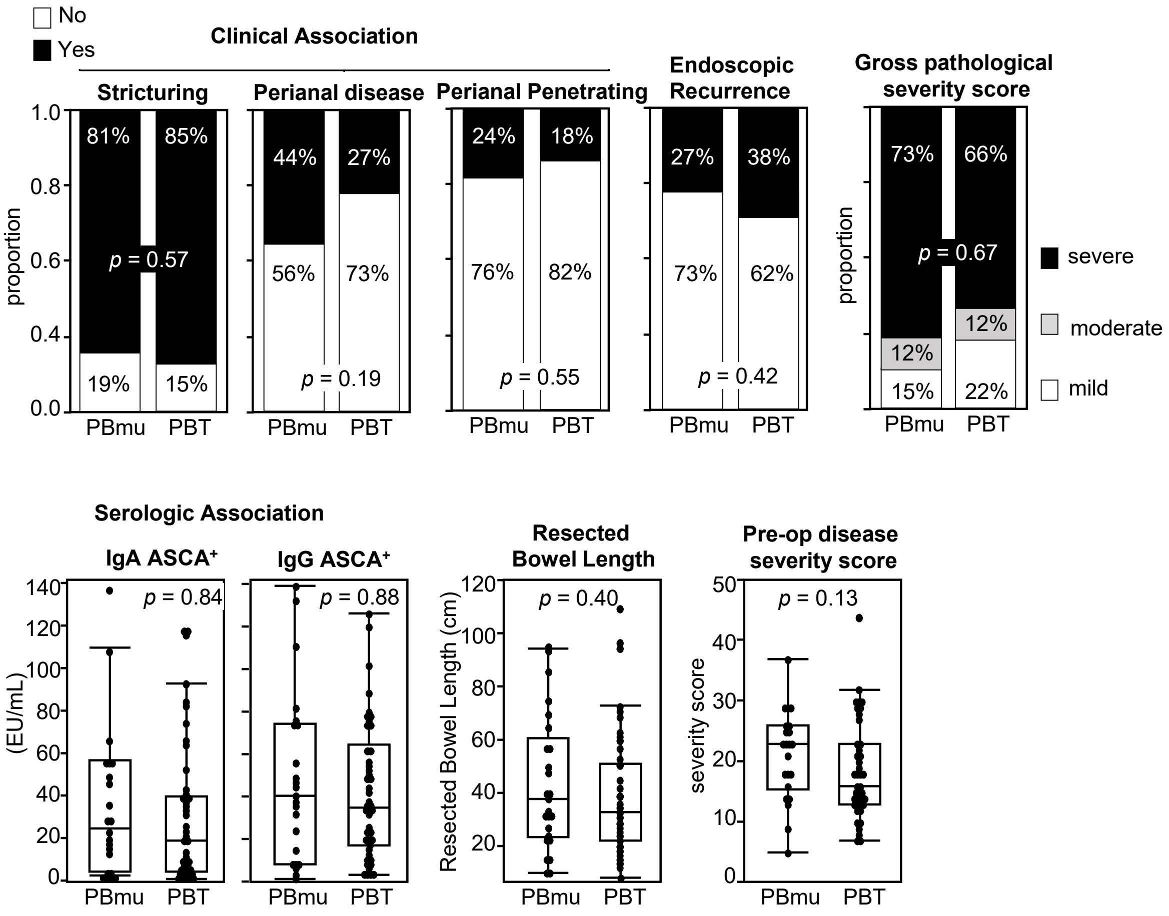

Figure S2: Transcriptomic signature stratifying CD-PBmu vs CD-PBT subtypes was not associated with clinical or serological indicators of disease burden.

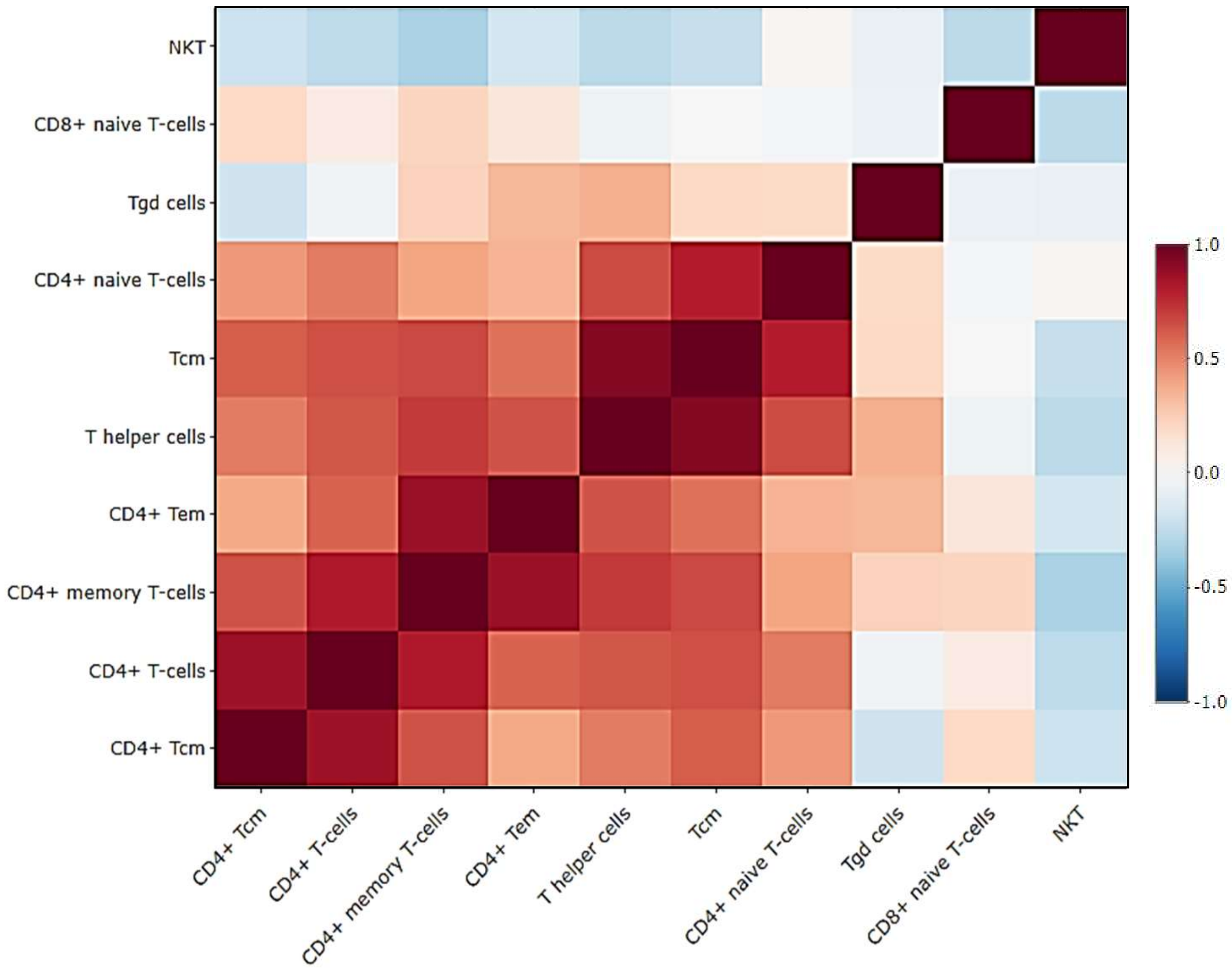

Figure S3: Correlation Matrix plot between the CD PBmu NKT and CD4$^+$/CD8$^+$ T cell subset enrichment scores. There is no significant positive or negative correlation between NKT with CD4$^+$/CD8$^+$ cell enrichment scores.

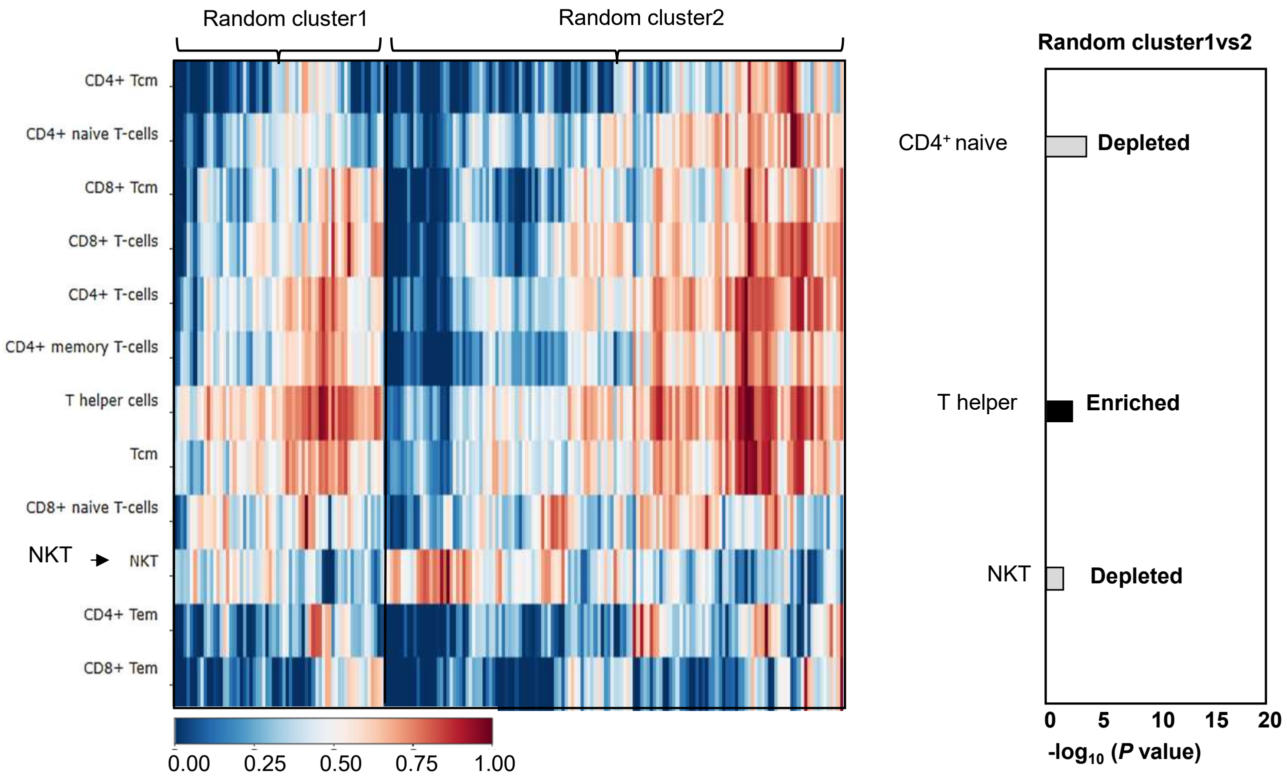

Figure S4: Heatmap based on cellular enrichment scores using xCell bioinformatics tool. Clusters were generated using a random gene probe set as input.

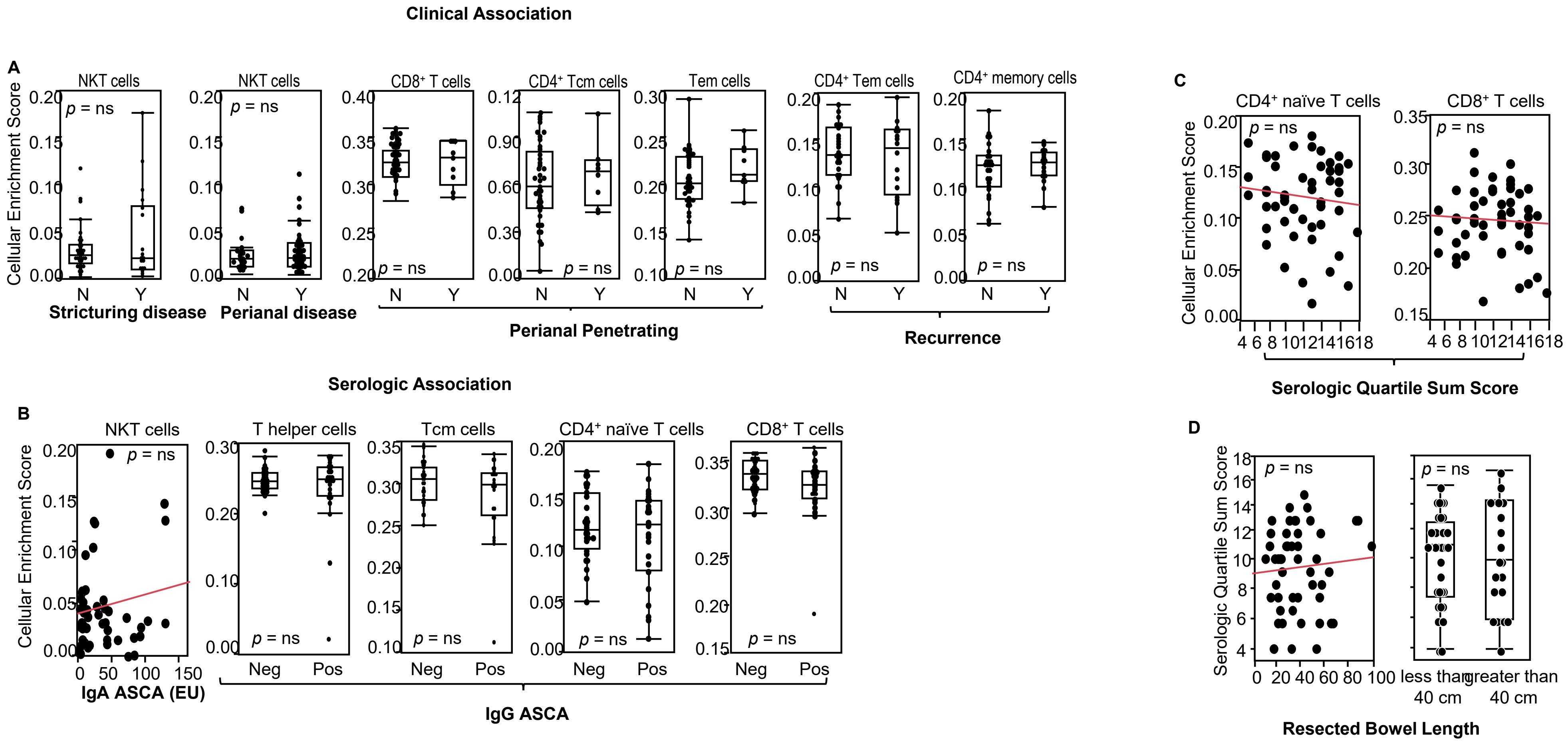

Figure S5: CD-PBT T cell subset composition is not associated with clinical and serological parameters of complicated disease (**A**) Association of NKT enrichment with stricturing disease and perianal disease and CD4⁺/CD8⁺ T cell subset depletion in CD-PBT with and perianal penetrating disease and post-operative endoscopic recurrence (N= Rutgeerts score 0-1; Y=2-4) (**B**) Association of NKT enrichment and CD4⁺/CD8⁺ T cell subset depletion in CD-PBT with ASCA seropositivity. (**C**) Inverse correlation of serological quartile sum scores in CD-PBT with of CD4⁺/CD8⁺ T cell subsets depletion. (**D**) Association of serological quartile sum scores in CD-PBT with increased length of bowel resection.

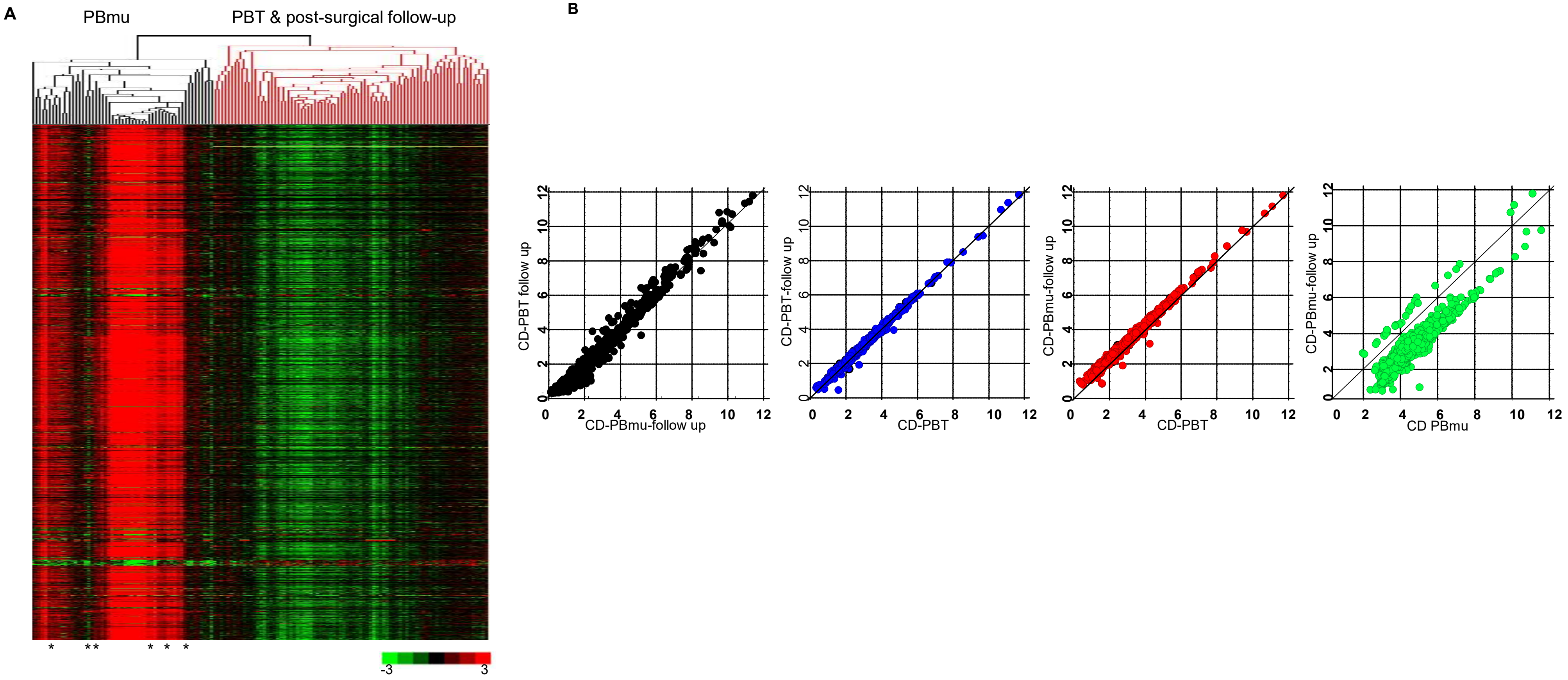

Figure S6: CD-PBmu gene expression profile reverts to that of CD-PBT following surgery. (A) Hierarchical clustering and heatmap of the 1566 genes defining the CD-PBmu and CD-PBT subtypes comparing peripheral CD3$^+$ T cell expression in all samples prior to surgery and post-operatively. Asterix denotes samples that did not cluster as predicted. **(B)** Scatter plot shows high correlation of gene expression between CD-PBmu subtype samples following surgery and CD-PBT subtype pre- or post surgery.

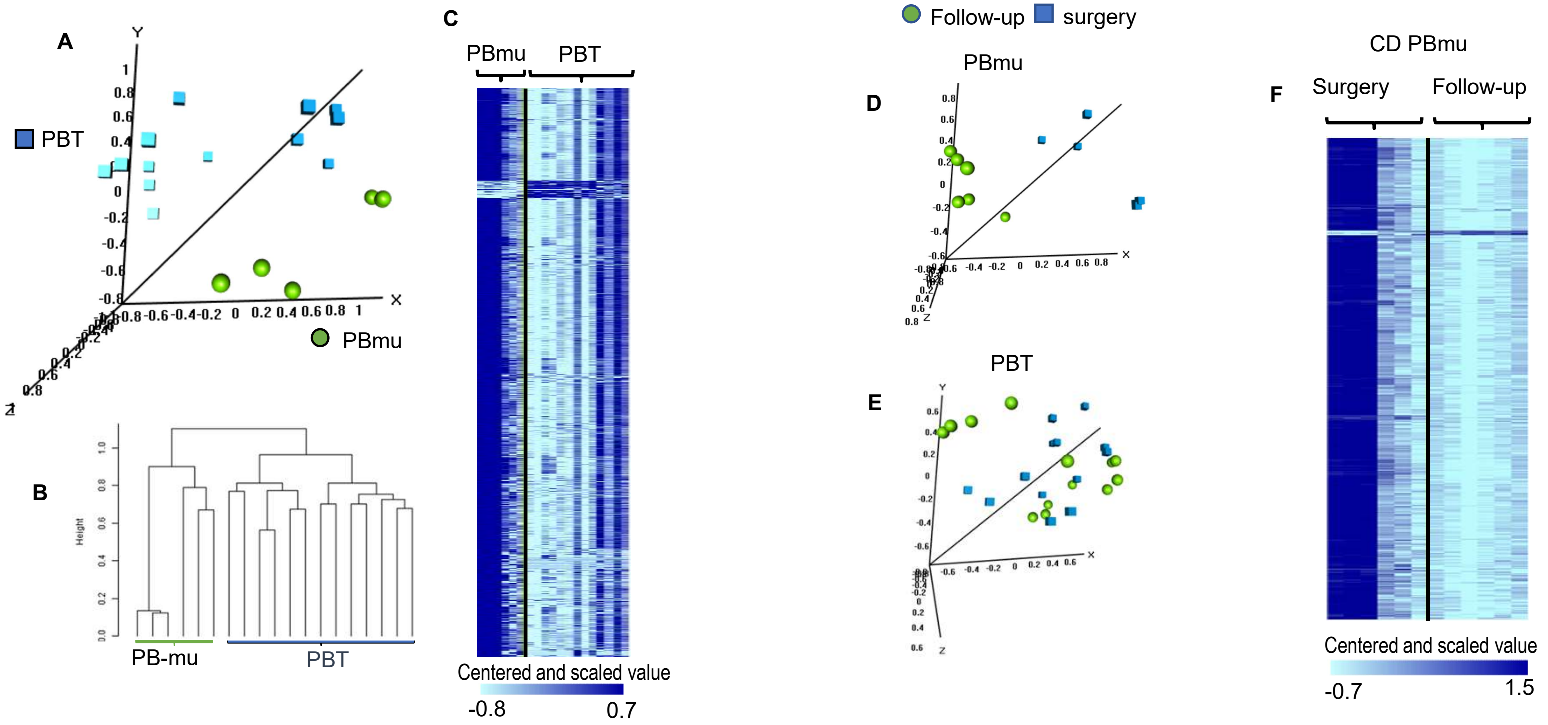

Figure S7: Validation of CD-PBmu gene signature reversion following surgery in a cohort of subjects comparing samples isolated at time of surgery to post-operative samples from same individuals (n=19). (A) PCA and **(B)** Hierarchical clustering of samples at time of surgery. **(C)** Heatmap of expression data for the same genes defining the CD-PBmu and CD-PBT subtypes in Figure 1. **(D)** PCA analysis of samples at surgery and post-operatively for CD-PBmu or **(E)** CD-PBT **(F)** Heatmap of expression data from genes previously defined in CD-PBmu samples pre- and post-surgery in Figure 2 (624/901 genes were differentially expressed p value <0.05). No genes were differentially expressed in CD-PBT when comparing pre to post surgery.

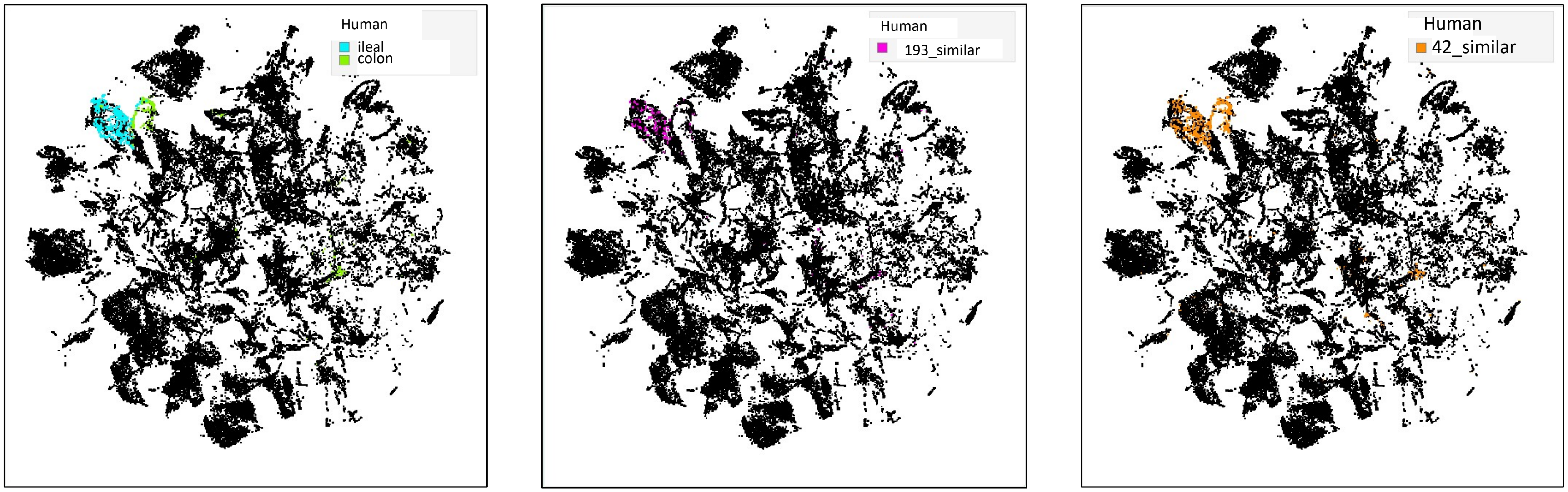

Figure S8: A CD-PBmu peripheral gene signature shows similar co-expression with ileal/colonic tissue. ARCHS4 generated t-SNE plots of gene signature from differentially up-regulated gene panel in CD-PBmu versus CD-PBT overlaps with similar co-expression from ileal and colonic tissue.

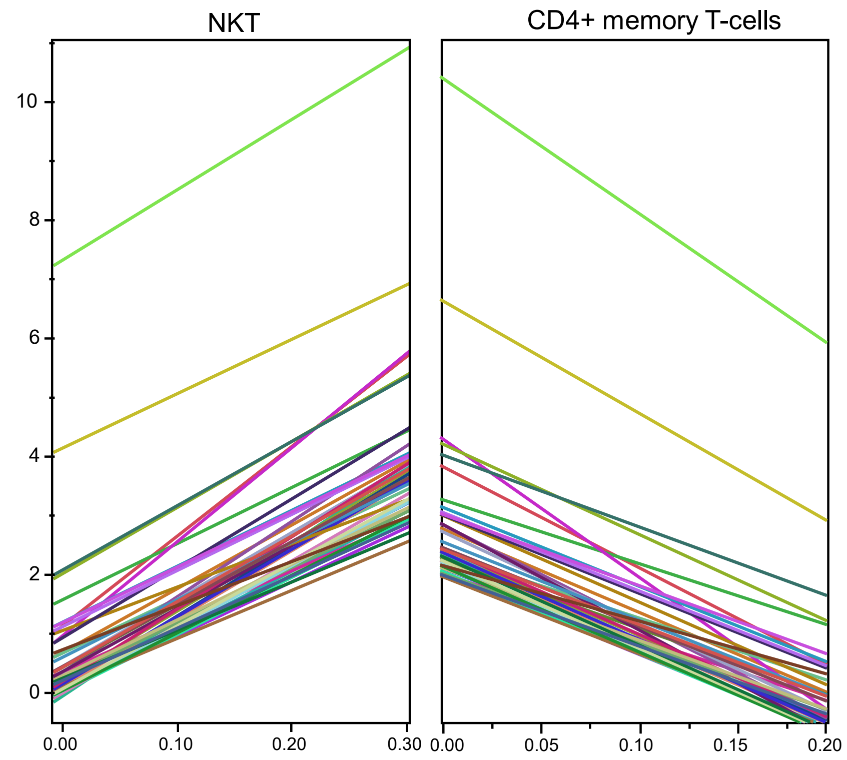

Figure S9: Correlation plot of biomarker gene panel expression versus enrichment scores for NK T cell and CD4+ memory T cell subsets.

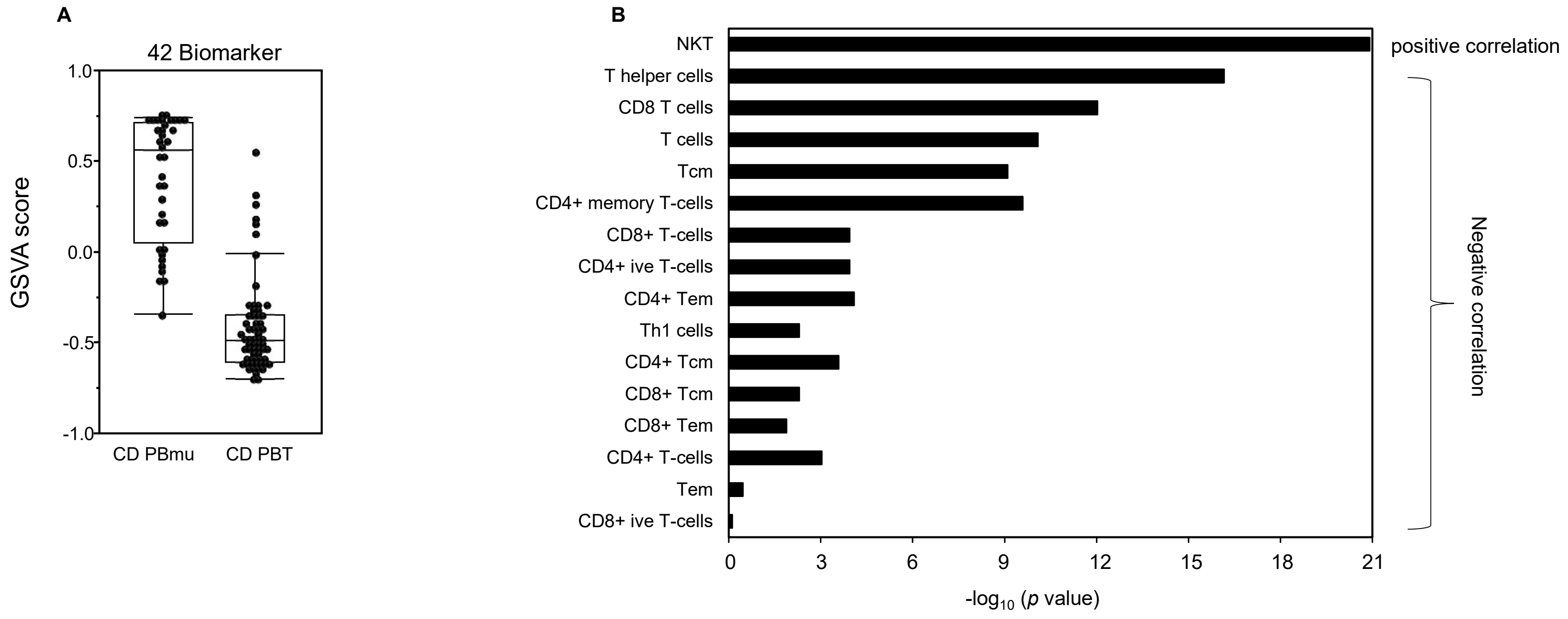

Figure S10: Gene Set Variation Analysis (GSVA) scores for the 42-biomarker gene panel. A. CD-PBmu vs CD-PBT GSVA scores are elevated in CD PBmu and B. display a positive correlation with NKT and negative correlation with T cell subset enrichment scores.

A

Classifier:	% correct classification	Sensitivity	Specificity	PPV	NPV
Compound Covariate Predictor	85	0.61	0.98	0.95	0.82
Diagonal Linear Discriminant Analysis	86	0.62	0.98	0.95	0.83
1-Nearest Neighbor	93	0.83	0.94	0.89	0.91
3-Nearest Neighbor	92	0.82	0.94	0.89	0.90
Nearest Centroid	85	0.61	0.98	0.94	0.82
Support Vector Machine	94	0.84	0.94	0.89	0.94
Bayesian Compound Covariate	89	0.46	0.86	0.63	0.74

B

Classifier:	% correct classification	Sensitivity	Specificity	PPV	NPV
Compound Covariate Predictor	90	0.77	0.95	0.87	0.90
Diagonal Linear Discriminant Analysis	89	0.75	0.94	0.86	0.89
1-Nearest Neighbor	94	0.82	0.95	0.88	0.92
3-Nearest Neighbor	92	0.78	0.95	0.87	0.90
Nearest Centroid	91	0.80	0.95	0.88	0.91
Support Vector Machine	93	0.82	0.94	0.86	0.92
Bayesian Compound Covariate	95	0.65	0.83	0.63	0.84

Table S1: Performance of cross-validation of CD-PBmu vs CD-PBT classification (**A**) CD-PBmu vs CD-PBT classifiers during cross-validation (**B**) CD-PBmu transcriptomic signature in classifying of whole blood validation cohort (GSE100833) into PBmu-like and PBT-like patient subtypes.
Positive Predictive Value (PPV), Negative Predictive Value (NPV)

Patient characteristics at time of surgery		Total	PBmu	PBT
**Number of patients**		n=100	n=36	n=64
**Variable n/total (%)**				
**Gender**	**Female**	41/100 (41)	11/36 (31)	30/64 (47)
**Age at diagnosis (median and IQR), yr.**		24 (16-32)	25 (18-35)	23 (16-32)
**Montreal classification**				
≤16 years (A1)		25/97(26)	6/35 (17)	19/62 (31)
17-40 years (A2)		55/97(57)	23/35 (66)	32/62 (52)
>40 years (A3)		17/97(18)	6/35 (17)	11/62 (18)
**Disease duration (median and IQR), yrs.**		7 (3-14)	8 (3-16)	7 (2-13)
Age at surgery (median and IQR), yr.		35(24-51)	37(27-53)	35(24-49)
Family history of IBD		34/97 (35)	10/35 (29)	24/62 (39)
First resection		63/86 (73)	22/29 (75)	41/57 (72)
Anemia at surgery		37/73 (51)	11/24 (46)	26/49 (53)
Elevated CRP at surgery		44/74 (60)	15/27 (56)	29/47 (62)
pre-op overall severity index (median and IQR)		15 (12-22)	19 (14-24)	14 (10-20)
**Pre-operative treatment history**				
Steroids		70/91 (77)	27/31 (87)	43/60 (71)
anti-TNF		58/87 (67)	16/27 (59)	42/60 (70)
immunomodulators		62/77 (81)	21/25 (84)	41/52 (79)
**CD disease location**				
L1 (ileal)		6/97 (6)	4/34 (11)	2/58 (3)
L2 (colonic)		11/97 (11)	3/34 (9)	8/58 (13)
L3 (ileocolonic)		80/97 (83)	28/34 (80)	52/58 (84)
**CD Disease Behavior**				
B1 (non-stricturing, non-penetrating)		13/89 (15)	4/29 (14)	9/60 (15)
B2 (isolated stricturing)		48/89 (54)	16/29 (55)	32/60 (53)
B3 (penetrating or stricturing and penetrating)		28/89 (31)	9/29 (31)	19/60 (32)
Perianal disease		24/73 (33)	11/24 (46)	13/49 (25)
**Resected Disease location**				
Small bowel		6/99 (6)	3/36 (8)	3/63 (5)
Ileocecal/Ileocolic		66/99 (67)	21/36 (58)	45/63 (71)
Colon		27/99 (27)	12/36 (34)	15/63 (24)
Resected bowel length (median and IQR), cm.		33 (22-56)	38 (24-61)	33 (22-51)
Granuloma in resected segment		26/77 (34)	8/25 (32)	18/52 (35)
Microscopic disease at margins		17/76 (22)	8/25 (32)	9/51 (18)
**Severity Score**				
pre-op overall severity index (median and IQR)		15 (12-22)	19 (14-24)	14 (10-20)
Pathology based severity score - deep ulcers, stricturs, fistula, fissures				
mild activity (1-2.9 cm)		19/94 (20)	5/34 (15)	14/60 (23)
moderate activity (3-5 cm)		11/94 (11)	4/34 (12)	7/60 (12)
severe activity (>5 cm, deep fissures		64/94 (68)	25/34 (74)	39/60 (65)
**post-op endoscopic recurrnace (Rutgeerts score)**				
Time between resection and colonoscopy (median and IQR), mo		10 (7-23)	10 (6-20)	10 (7-25)
I0		20/75 (27)	7/26 (27)	13/49 (27)
I1		23/75 (31)	9/26 (35)	14/49 (29)
I2		9/75 (12)	2/26 (8)	7/49 (14)
I3		12/75 (16)	3/26 (12)	9/49 (18)
I4		1/75 (1)	1/26 (4)	0/49 (0)
no post-op endoscopy		10/75 (13)	4/26(15)	6/49 (12)
**post-op prophylactic medication**				
Immunmosuppresants		32/63 (51)	10/22 (46)	22/41 (54)
anti-TNF		39/64 (61)	10/22 (46)	29/42 (69)

Table S3

comparison	overall p-value	overall z-score	UR (p-value)	DE (p-value)	UR (z-score)	DE (z-score)
NK T cell vs naïve CD8+ T cell	12.47	21.38	7.12E-06	9.38E-20	35.04	50.49
NK T cell vs naïve CD4+ T cell	8.55	19.2	5.41E-08	9.76E-09	39.74	37.05
NK T cell vs CD4+ effector memory T cell	3.46	8.11	1.30E-05	0.04	32.44	
NK T cell vs CD4+ central memory T cell	2.63	7.4	1.31E-04		29.62	
NK T cell vs CD8+ central memory T cell	1.4		0.03			

Table S4: Concordance of CD-PBmu signature matching gene expression and upstream regulatory pathways associated when comparing NKT cell to CD4 T cell subsets (Geo accession:GSE24759).

653 samples from Ileal biopsies from treatment-naïve pediatric CD patient studies:					
Accession ID	Title	Contributor	cohort	Similar co-expression	Citation
GSE62207	Ileal immune maturation in Pediatric Crohn's Disease	Haberman Y, Denson L, Karns R	310 treatment-naïve pediatric Crohn Disease patients and controls.	216 CD patients<br>32 non-IBD patients	No citation
GSE57945	Core Ileal Transcriptome in Pediatric Crohn Disease	Haberman Y, Denson L, Karns R	359 treatment-naïve pediatric Crohn Disease, Ulcerative Colitis patients and controls.	177 CD patients<br>36 UC patients<br>27 non-IBD patients	J Clin Invest 2014;124(8):3617-33.<br>Sci Rep 2019 7;9(1):16163
GSE93624	Profiling of Ileal Transcriptome in Pediatric Crohn Disease	Marigorta UM, Gibson G, Kugathasan S, Denson LA	210 treatment-naïve patients of pediatric Crohn's disease and 35 non-IBD controls	146 CD patients<br>19 non-IBD patients	Nat Genet 2017 Oct;49(10):1517-1521.
272 samples from IBD studies:					
GSE83687	A functional genomics predictive network model identifies regulators of inflammatory bowel disease: Mount Sinai Hospital (MSH) Population Specimen Collection and Profiling of Inflammatory Bowel Disease	Contact name: Eric Schadt	134 patients undergoing bowel resection for inflammatory bowel disease (IBD) and non IBD controls at Mount Sinai Medical Center were collected as the source of tissue.	35 CD patients<br>24 UC patients<br>49 non-IBD control	Nat Genet 2017;49(10):1437-1449.
GSE81266	Ileal pouch transcriptomics reveal shared pathogenesis between pouchitis and ulcerative colitis	Huang Y, Dalal S, Chang EB	biopsies of 17 UC pouch and prepouch ileum were collected within 12 months after IPAA surgery and 4 patients with FAP	18 UC patients (60 samples, 1UC patient with pouch sample only)<br>2 FAP (3 samples), 1 non-IBD	Inflamm Bowel Dis 2017;23(3):366-378.
GSE72819	Gene expression of baseline biopsies from etrolizumab-treated ulcerative colitis (UC) patients	Keir ME, Tew GW, Hackney JA, Gibbons D et al.	Baseline colonic biopsies from 110 UC patients in a phase 2 placebo-controlled trial of etrolizumab, and from 21 patients with UC or without IBD.	Anti-TNF naïve: 18 non-remitter, 8 remitter<br>Prior anti-TNF: 38 non-remitter, 3 remitter, 3 N/A	Gastroenterology 2016;150(2):477-87.e9.
GSE66207	mRNA and small RNA associated with Crohn's disease behavior [RNA-Seq]	Contact name: Bailey Peck	33 Macroscopically non-inflamed colon tissue from well-characterized Crohn's disease patients and normal controls.	6 non-stricturing, non-penetrating<br>5 structuring; 8 penetrating<br>11 (cancer, diverticulitis, other)	Inflamm Bowel Dis 2015 Sep;21(9):2178-87

Table S5: Source of overlapping bowel tissue with similar co-expression to CD-PBmu and 42 biomarker gene signatures
